# Caregivers’ experiences of caring for children with developmental dysplasia of the hip: a qualitative descriptive review

**DOI:** 10.3389/fmed.2026.1772584

**Published:** 2026-05-14

**Authors:** Lei Yang, Lingyan Fan, Yan Jiang, Wenjuan Shao, Weili Shen, Qianyi Xiao

**Affiliations:** Department of Orthopedics, Shanghai Children’s Hospital, School of Medicine, Shanghai Jiao Tong University, Shanghai, China

**Keywords:** caregivers, children, developmental dysplasia of the hip, parenting experiences, qualitative review

## Abstract

**Background:**

Developmental dysplasia of the hip (DDH) is a common pediatric orthopedic condition. Caregivers of children with DDH often face significant challenges that may affect their quality of life. This review aimed to systematically identify, appraise, summarize the available qualitative evidence on caregivers’ experiences of caring for children with DDH.

**Methods:**

We systematically searched eight databases (Embase, Cochrane Library, Web of Science, PubMed, CINAHL, PsycINFO, ERIC, and ProQuest) from inception to May 2025. Two researchers independent screened studies, extracted data, and assessed methodological quality using the Joanna Briggs Institute critical appraisal checklist for qualitative research. A meta-aggregative approach was used to categorize and summarize themes reported in the primary studies. Confidence in each summary finding was assessed using GRADE-CERQual.

**Results:**

Eight studies met the inclusion criteria. These included four interview-based studies (total 66 participants) and four survey-based studies with open-ended questions (total 1,522 participants). Four main themes were identified: (1) a challenging and often delayed diagnostic process; (2) treatment devices complicate daily parenting tasks; (3) inadequate or ill-timed informational and practical support; and (4) gradual adaptation and skill development over time.

**Conclusion:**

Caregivers of children with DDH face substantial challenges during diagnosis, treatment, and daily care. The findings highlight the potential value of timely, practical, and empathetic support that addresses informational needs and acknowledges the adaptive work families undertake. Future research should employ more in-depth qualitative designs to capture the nuanced processes of caregiver adaptation.

## 1 Introduction

Developmental dysplasia of the hip (DDH) is a condition characterized by abnormal development of the acetabulum and proximal femur. Its reported incidence ranges from 1/1,000 to 20/1,000, varying with geographic location, population background, and diagnostic criteria (1). An ultrasound screening study in China reported a DDH detection rate of 3.2% (196/6189), with a predominance of left-sided involvement (64.73%) (2). Additionally, a study in Saudi Arabia found the incidence of DDH ranged from 3.1 to 4.9 per 1,000 births, with prevalence varying between 6 and 78% (3). DDH shows a female and left-side predominance and, if untreated, can lead to chronic pain, functional impairment, and secondary arthritis (4). Treatment is age-dependent, with non-surgical interventions such as the Pavlik harness preferred in early infancy. Surgical reduction is required for missed or refractory cases (5).

Recent studies indicate that caregivers of children with DDH often face challenges due to insufficient knowledge about disease during the caregiving process (6) and commonly experience significant emotional burden (7). Caregivers’ anxiety is particularly prone to being triggered when receiving diagnosis, managing brace discomfort, worrying about functional recovery, and concerning their child’s future walking ability (8). The timing of diagnosis influences caregivers’ focus of concern; those with early diagnosis tend to worry more about daily care difficulties, while those with later diagnosis are more concerned about long-term prognosis (8, 9). The caregiving experience also varies depending on the treatment method (10, 11). The extended treatment and follow-up process for DDH poses challenges to the quality of life of caregivers, often accompanied by physical and mental exhaustion (7). Simultaneously, caregivers frequently experience social isolation due to a lack of social understanding (7), while also facing economic pressure and adjustments in family roles (10). Research indicates that main obstacles during caregiving are the lack of practical caregiving resources and consistent medical guidance (6, 7).

Although existing qualitative studies has revealed various aspects of caregivers’ experiences (7, 10), findings come from diverse contexts with varying methodological rigor. Currently, no systematic review has comprehensively identified and summarized this body of qualitative evidence. Therefore, we conducted a qualitative systematic review to identify, appraise, and summarize experiences of caregivers of children with DDH, using a meta-aggregative approach to produce a descriptive summary that may inform future research and practice.

## 2 Methods

### 2.1 Aims

This study employed a qualitative meta-aggregative approach to systematically identify, appraise, and summarize the findings of qualitative research on the experiences of primary caregivers of children with DDH. The review addressed three questions: (1) What difficulties were encountered in taking care of children with DDH? (2) What strategies did parents/caregivers use to address these difficulties? (3) What are the effects of disease treatments on children and parents/caregivers?

### 2.2 Protocol registration

This study was conducted according to the International Prospective Register of Systematic Reviews (PROSPERO) publication study protocol (no. 42022301235). Our research was reported following the PRISMA (Preferred Reporting Items for Systematic Reviews and Meta-Analyses) and the ENTREQ (Enhancing Transparency in Reporting the Synthesis of Qualitative Research) statements.

### 2.3 Inclusion criteria

The study had to meet the following criteria: the study population (P) comprised primary caregivers of children with DDH; the phenomena of interest (I) included the feelings, stress load, challenges, and needs of primary caregivers when caring for children with DDH; and the context (Co) was any hospital or caregiver’s home. This study included articles that covered qualitative research, including mixed-methods studies where the qualitative data could be separated. Furthermore, articles with incomplete texts, studies that adopted mixed methods in which qualitative data could not be separated, quantitative research, exploratory studies, reviews, and conference abstracts were rejected.

### 2.4 Search strategy

The first reviewer (YL) performed a comprehensive and systematic literature search using the following electronic databases: Embase, Cochrane Library, Web of Science, Pubmed (MEDLINE), CINAHL, PsycINFO, ERIC and ProQuest. The search period was from database establishment until May 2025. First, we performed a preliminary retrieval from PubMed to determine the keywords of the relevant articles. The keywords were the following: “developmental dysplasia of the hip/developmental hip dislocation/congenital hip dislocation/DDH”, “father/mother/parents/caregiver/carer/family/informal carers/relatives/paternal”, and “experience/perceptions/attitudes/views/feelings/caring/nursing/parenting.” Based on the preliminary search and under the guidance of library staff, we formulated a corresponding search strategy according to the grammatical rules of each database. The full PubMed search strategy is provided in Supplementary Material S1. The search results were then imported into Endnote V.X9 (Clarivate Analytics, Philadelphia, Pennsylvania, USA), which was used to organize the literature, and further studies were examined independently.

### 2.5 Study selection

**Figure 1:**
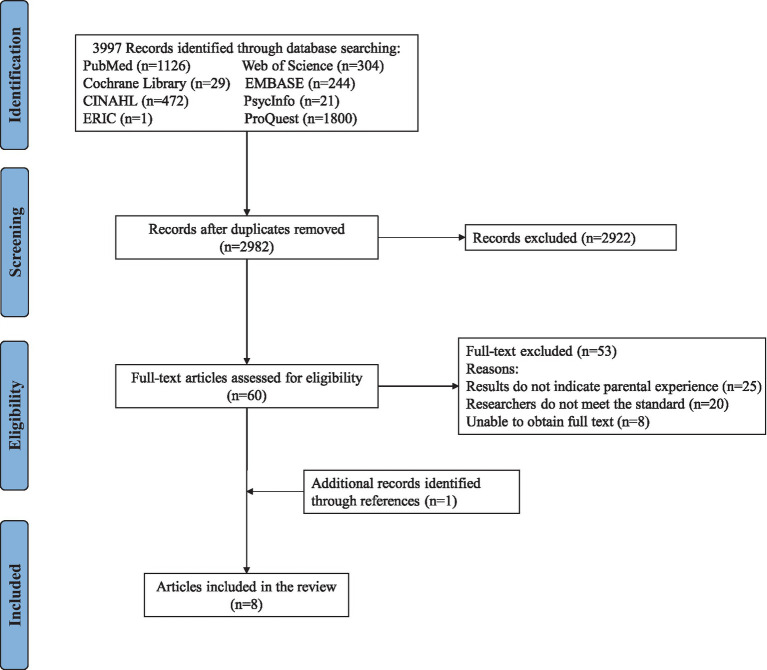
PRISMA flow diagram.

After completing the literature search, all search results were imported into EndNote V.X9. First, one reviewer (YL) removed the duplicates. Two independent reviewers (JY and SWJ) screened the titles, abstracts, and full texts of potential studies to ensure that they met the inclusion criteria. Figure 1 illustrates the different phases of the review. When disagreements arose, the reviewer made judgments through discussion or consultation with a third researcher (FLY). The interrater reliability test was performed, and we aimed to achieve at least moderate agreement with a Cohen’s K > 0.4 (12). The Cohen’s K value in this study was 0.714.

### 2.6 Assessment of methodological quality

The Joanna Briggs Institute critical appraisal checklist (13) for qualitative research was applied independently by two researchers to evaluate the quality of the included articles. The checklist consists of 10 entries, each reflecting the key aspects of rigor, credibility, conceptual depth and methodology. The criteria for all questions were scored as ‘yes’, ‘no’, or ‘unclear’. All included studies were critically evaluated without regard to quality and were considered potentially valuable because although some studies only represented specific cultural areas, exclusion would have narrowed the scope of the analysis. Therefore, while integrating the results, we considered the evaluation score. The results of the critical evaluation are presented in Table 1.

**Table 1:** Results of quality assessment based on the Joanna Briggs Institute critical appraisal checklist for qualitative studies.

Studies	C1	C2	C3	C4	C5	C6	C7	C8	C9	C10
1. Chao and Chiang (2003) (16)	Y	Y	Y	U	Y	N	N	Y	U	Y
2. Gibbard et al. (2021) (6)	U	Y	Y	N	Y	N	Y	Y	U	Y
3. Grzybowski et al. (2023) (18)	U	Y	Y	N	Y	N	Y	Y	U	Y
4. Harry et al. (2022) (7)	Y	Y	Y	Y	Y	N	Y	Y	Y	Y
5. Poole (2019) (17)	Y	Y	Y	Y	Y	Y	Y	Y	Y	Y
6. Theunissen et al. (2022) (11)	Y	Y	Y	Y	Y	N	Y	Y	U	Y
7. Theunissen et al. (2023) (19)	Y	Y	Y	Y	Y	N	Y	Y	U	Y
8. Wakely et al. (2021) (10)	Y	Y	Y	N	Y	N	Y	Y	Y	Y

C1 = Congruity between the stated philosophical perspective and the research methodology. C2 = Congruity between the research methodology and the research question or objectives. C3 = Congruity between the research methodology and the methods used to collect data. C4 = Congruity between the research methodology and the representation and analysis of data. C5 = There is congruence between the research methodology and the interpretation of results. C6 = Locating the research culturally or theoretically. C7 = Influence of the researcher on the research. C8 = Representation of participants and their voices. C9 = Ethical approval by an appropriate body. C10 = Relationship of conclusions to analysis, or interpretation of the data. Y, yes; N, no; U, unclear.

### 2.7 Data extraction

Based on methodological quality assessment, the first reviewer searched for relevant information in the included articles. Date regarding the author, publication year, country, design (data gathering technique), phenomenon of interest, recruitment and participation, and principal results with illustrations. Each finding was then assigned a confidence level of “unequivocal,” “credible,” or “unsupported” (13).

### 2.8 Data synthesis

The data synthesis aimed to integrate the extracted results into a meaningful set of statements that allowed for the explicit identification of themes appearing in the data. The meta-aggregation process was guided by the Jonna Briggs Institute System for the Unified Management, Assessment, and Review of Information (JBI-SUMARI) (14), which procedure involved four steps: (1) Reviewers carefully examined and extracted key viewpoints; (2) Reviewers assigned a credibility rating to each finding; (3) Data were categorized and synthesized, with findings rated as “unsupported” excluded from further analysis; (4) By comparing the similarities and differences across categories, similar categories were merged into higher-level analytical themes. These analytical themes represent summary categories of the original study findings and do not purport to generate new theoretical interpretations beyond the original authors’ claims.

### 2.9 Confidence in the findings

To assess the strength of the evidence for our review findings, we applied the GRADE-CERQual (Grading of Recommendations Assessment, Development and Evaluation-Confidence in Evidence from Reviews of Qualitative research) tool (15). For each synthesized finding, we evaluated four key components: methodological limitations, coherence, adequacy and relevance. Based on assessment of these components, we graded our overall confidence in each finding as high, moderate, low, or very low, and the results were presented in Table 2. It is important to distinguish between the JBI credibility ratings (applied to individual extracted findings from primary studies) and the GRADE-CERQual confidence ratings (applied to the synthesized review findings).

**Table 2:** GRADE-CERQual summary of findings.

Theme	Contributing studies	Methodological limitations	Relevance	Coherence	Adequacy	Overall confidence
(1) A challenging and often delayed diagnostic process	Gibbard et al. (2021) (6), Theunissen et al. (2022) (11), Chao and Chiang (2003) (16), Poole (2019) (17), Harry et al. (2022) (7)	Moderate concerns^1^	No concerns	No concerns	Moderate concerns^2^	Moderate
(2) Treatment devices complicate daily parenting tasks	Theunissen et al. (2022) (11), Wakely et al. (2021) (10), Grzybowski et al. (2023) (18), Harry et al. (2022) (7), Poole (2019) (17)	Moderate concerns^1^	No concerns	No concerns	Moderate concerns^2^	Moderate
(3) Inadequate or ill-timed informational and practical support	Gibbard et al. (2021) (6), Theunissen et al. (2022) (11), Wakely et al. (2021) (10), Harry et al. (2022) (6), Poole (2019) (17)	Moderate concerns^1^	No concerns	No concerns	Moderate concerns^2^	Moderate
(4) Gradual adaptation and skill development over time	Chao and Chiang (2003) (16), Wakely et al. (2021) (10), Poole (2019) (17), Theunissen et al. (2023) (19)	Severe concerns^3^	No concerns	Minor concerns^4^	Severe concerns^5^	Low

^1^Some contributing studies used survey-based qualitative data with limited depth; Chao and Chiang (2003) (16) is a single-case study. ^2^Although multiple studies reported this theme, the number of in-depth interview studies is limited (*n* = 2–4). ^3^This theme relies heavily on one in-depth case study [Chao and Chiang (2003) (16)] with methodological limitations (e.g., lack of researcher reflexivity, unclear ethical approval, single case). ^4^While coping and adaptation are consistently reported, the specific mechanisms of gradual adaptation and skill development are more deeply elaborated in Chao and Chiang (16) than elsewhere. ^5^Data adequacy is severely limited, relying primarily on a single case study.

### 2.10 Handling of data heterogeneity

The studies included in this review exhibited heterogeneity in design, including in-depth interview studies and open-ended questionnaire surveys. We recognize that these two data types differ significantly in data richness. During the synthesis, we adopted the following strategies: (1) clearly indicating the primary evidence source (interview/survey) for each theme in the Results section; (2) downgrading the adequacy rating in GRADE-CERQual assessments when findings were primarily based on survey data; (3) discussing the impact of this heterogeneity on the confidence of findings in the Limitations section of the Discussion.

## 3 Results

### 3.1 Study characteristics

The study characteristics of the 8 articles included in this study are listed in Table 3. Various qualitative study designs were used, including phenomenological (*n* = 4), qualitative (*n* = 3) and no specified (*n* = 1). Different methods were used for data collection, including semi-structured interviews (*n* = 4), and open-ended questionnaire (*n* = 4). The majority of the listed research was published between 2019 and 2025 (*n* = 7). The research was based in five countries: Canada (*n* = 2), Australia (*n* = 2), the Netherlands (*n* = 2), China (*n* = 1). It should be noted that two studies collected both quantitative survey data and qualitative open-ended responses. Only the qualitative data from these studies were extracted and synthesized. Consequently, the evidence base comprised four interview-based studies (total 66 participants) and four survey-based qualitative studies (total 1,522 participants). Two studies contributed both quantitative and qualitative data, but only qualitative portions were included.

**Table 3:** Characteristics of the studies.

Study (year), country	Design	Qualitative data type	Data collection method	Phenomenon of interest	Recruitment and participants	Main findings
Chao and Chiang (2003) (16), China	Not specified	Case study	Semi-structured interview (*n* = 1 caregiver)	Impact on and coping of mothers	One 32-year-old Chinese mother	Five categories: shock, fear, loss/anger, uncertainty, excessive emotions
Gibbard et al. (2021) (6), Canada	Descriptive qualitative	Thematic analysis	Open-ended questionnaire (*n* = 739 caregivers)	Patient/family experiences across diagnosis and treatment	International online survey	Delayed diagnosis, lack of info/resources, emotional burden, value of online communities
Grzybowski et al. (2023) (18), Canada	Descriptive qualitative	Thematic analysis	Open-ended questionnaire (*n* = 530 caregivers)	Caregiver experiences with DDH orthotics	Online survey from 20 countries	Comfort/irritation, ease of use, cleanliness, daily activity impediment
Harry et al. (2022) (7), Australia	Descriptive qualitative	Thematic analysis	Open-ended questionnaire (*n* = 753 caregivers)	Effect of DDH management on parenting	Online survey in Australia	Arduous parenting, insufficient understanding, inconsistent guidance
Poole (2019) (17), the UK	Interpretative phenomenological analysis	IPA	Semi-structured interviews (*n* = 18 parents: 9 mothers, 9 fathers)	Experiences of parents of infants with DDH	DDH charity social media	Mothers: empowerment/disempowerment, relationship dynamics; Fathers: managing disrupted family
Theunissen et al. (2022) (11), Netherlands	Phenomenology	Thematic analysis	Semi-structured interviews (*n* = 20 parents)	Experiences of caring for a child with DDH treated with Pavlik harness	Máxima Medical Centre	Positive experiences with professionals, insufficient information, treatment concerns, parenting difficulties, emotional burden
Theunissen et al. (2023) (19), Netherlands	Phenomenology	Thematic analysis	Semi-structured interviews (*n* = 20 parents)	Information needs of parents during diagnosis and treatment	Máxima Medical Centre	General information, patient-specific information, practical information, future perspectives
Wakely et al. (2021) (10), Australia	Phenomenology	Thematic analysis	Semi-structured interviews (*n* = 7 parents: 6 mothers, 1 father)	Lived experience of parenting a child with DDH	Australian health service	Surrendering the parenting prerogative, struggling to adjust day-to-day

### 3.2 Review findings

#### 3.2.1 Theme 1: a challenging and often delayed diagnostic process

Across all eight studies, caregivers consistently described the diagnostic phase as a period of significant ambiguity and distress. This experience was characterized by three common sub-themes.

##### 3.2.1.1 Sub-theme A: prevalence of perceived diagnostic delay

Caregivers described that DDH assessment frequently required multiple encounters before confirmation, and their concerns were sometimes not acted upon, creating a sense of being dismissed. For instance, in Theunissen et al.’s (11) interview study, a parent reported being told their child’s symptoms were “growing pains.” This finding was echoed in the large-scale survey by Gibbard et al. (6), where many respondents indicated their concerns were dismissed by doctors. Another interview study (16) documented a mother’s self-blame for not seeking medical attention earlier.

##### 3.2.1.2 Sub-theme B: information overload and shock at diagnosis

When DDH was finally diagnosed, caregivers commonly reported shock, overwhelm, and confusion. These were not only emotional responses but also reflected information-processing strain at a moment when rapid decisions were expected. Participants in Theunissen et al. (11) described feeling “blown away” by specialists’ rapid technical explanations. Similar shock reactions were documented in Poole’s (17) phenomenological study and Chao and Chiang’s (16) case study.

##### 3.2.1.3 Sub-theme C: ongoing concerns about the child’s future

Caregivers’ insecurity extended beyond the diagnosis itself to anticipated functional outcomes and the possible need for surgery. Participants in Theunissen et al. (11) expressed persistent worries about whether their child would walk normally, whether legs would be equal in length, and whether sports participation would be possible. This worry manifested as a “future-oriented vigilance” rather than a single emotional reaction.

Evidence source note: This theme is primarily supported by interview-based studies (11, 16, 17) and large-scale surveys (6, 7). Interview data provided richer experiential details, while survey data confirmed the prevalence of these experiences.

#### 3.2.2 Theme 2: treatment devices complicate daily parenting tasks

Six studies reported substantial impacts of treatment devices (braces, harnesses, casts) on daily parenting. These impacts were not merely practical disruptions but reflected deeper shifts in parenting identity and parent–child relationships.

##### 3.2.2.1 Sub-theme A: technicalization of daily care tasks

Harnesses/braces turned everyday parenting tasks (holding, feeding, sleeping, clothing, hygiene) into technical and risk-laden procedures, increasing vigilance and anxiety about “doing it right.” In Wakely et al.’s (10) interview study, a mother described persistent anxiety during feeding about whether her infant’s hips were in the correct position. In Theunissen et al. (11), caregivers reported that holding their child felt “less personal,” with the infant feeling “like a parcel.”

##### 3.2.2.2 Sub-theme B: altered parent–child intimacy

Caregivers described reduced spontaneity and intimacy in holding and cuddling. A participant in Theunissen et al. (11) stated: “Especially the holding and cuddling. That felt less personal. It wasn’t really a baby anymore; it was more like a parcel.” Similar experiences were described in Wakely et al. (10).

##### 3.2.2.3 Sub-theme C: daily life disruption and role strain

The child’s dependence and frequent appointments meant caregivers struggled to sustain employment and routine household functioning. A mother in Theunissen et al. (11) described how her child’s continuous need for attention prevented her from doing household chores during the baby’s playtime. Social participation was also limited, partly due to time constraints and partly due to perceived risks of going out. Survey data from Grzybowski et al. (18) further quantified these difficulties: among 530 participants, most reported that the brace impacted daily activities, comfort, and cleanliness.

Evidence source note: This theme is supported by interview-based studies (10, 11) and surveys (7, 18). Survey data provided quantitative information on the prevalence of difficulties, while interview data revealed the emotional experience of these difficulties.

#### 3.2.3 Theme 3: inadequate or ill-timed informational and practical support

Caregivers’ narratives indicated that support failed in three specific ways: timing mismatch (support not available at the moment of highest uncertainty); content mismatch (generic reassurance vs. practical, actionable guidance); and credibility mismatch (difficulty judging online information quality).

##### 3.2.3.1 Sub-theme A: value of empathic listening and psychological support

Caregivers reported that empathic listening and validation supported coping. In Harry et al.’s (7) survey, a parent described how an older doctor who took time to listen to her concerns was helpful. Peer support was also highly valued—talking to others going through similar situations on Facebook support groups provided experiential understanding that formal services did not always offer.

##### 3.2.3.2 Sub-theme B: informational support: from scarcity to overload

Caregivers relied on the internet as a primary source but struggled to separate reliable information from alarming misinformation. A participant in Theunissen et al. (11) described receiving only brief notification of “severe” hip dysplasia at the diagnostic center, then searching online and encountering “the worst things,” which increased her worry. This suggests that “information support” is not merely access but curation and interpretive help, especially at diagnosis and treatment initiation.

##### 3.2.3.3 Sub-theme C: lack of economic support as a compound burden

Financial strain arose through both direct costs (adapted equipment, special clothing) and indirect income loss due to reduced work. In Wakely et al. (10), a mother detailed unexpected expenses for a new car seat, new clothes, and modified stroller, noting that health professionals did not adequately warn about these costs.

Evidence source note: This theme is supported by all eight studies. Rich descriptions of information overload and psychological support came primarily from interview studies (11, 19), while evidence for economic burden came mainly from Wakely et al. (10).

#### 3.2.4 Theme 4: gradual adaptation and skill development over time

Five studies reported that caregivers gradually adapted over time. Adaptation was not passive acceptance but an active process of rebuilding predictability through learning, skill development, and meaning-making.

##### 3.2.4.1 Sub-theme A: self-regulation and capability-building

Caregivers sought advice, reframed blame, and learned hands-on skills. In Chao and Chiang’s (16) case study, a mother, after being told that the leg length difference would be at most two centimeters, decided “don’t take all the blame” and committed to regular follow-up. The same mother actively learned how to change diapers: “I better learn it quickly right now to do the right things.”

##### 3.2.4.2 Sub-theme B: re-normalization of family life and meaning-making

Caregivers articulated a deliberate commitment to family cohesion and problem-solving. A participant in Chao and Chiang (16) stated: “What happened has happened. I have another boy to look after at home. Husband and wife need to think with the same heart. Not to argue with each other…. When there is a problem, face and think about how to solve it. Mum and Dad need to sit together, because this is our family, our home.”

##### 3.2.4.3 Sub-theme C: positive communication with the child

Caregivers described efforts to protect the child emotionally and sustain encouragement even under guilt and hardship. The mother in Chao and Chiang (16) said: “The boy is so poor to lie there all day with the cast all over him. This is all my fault. But I have to make him happier. It helps him to forget all the suffering he has now.”

Evidence source note: This theme is primarily supported by a single case study (16). Other studies (10, 17, 19) provided supporting but limited evidence. Consequently, confidence in this theme is severely limited.

#### 3.2.5 The GRADE-CERQual assessment for synthesized findings

The Table 2 presents the GRADE-CERQual assessment for each of the four themes. Confidence in each theme is judged based on methodological limitations of contributing studies, relevance to the review question, coherence across studies, and adequacy of data. Unlike the JBI credibility ratings used for individual extracted findings in primary studies, these GRADE-CERQual ratings reflect our overall confidence in each thematic finding of this review.

## 4 Discussion

This qualitative review summarizes caregiving experiences of caregivers of children with DDH across eight studies. Four main descriptive themes were identified: the challenging and delayed diagnostic process, the complication of daily parenting by treatment devices, inadequate informational and practical support, and gradual adaptation over time. Our findings align with recent work on family-centered outcomes for DDH. Craven et al. (9, 20) identified a family-centered core outcome set including infant comfort, feeding, sleep, cleanliness, parent–child bonding, and parental wellbeing. The diagnostic anxiety, brace-related difficulties, and economic burden reported by caregivers in this review directly map onto these outcome domains, validating their importance to families.

Diagnostic delay and information overload are primary sources of caregiver distress. This aligns with Gönen et al. (21), who highlighted the need for improved family physician knowledge and awareness of DDH screening. Zhang et al. (22) also noted that early diagnosis is crucial for improving prognosis. These findings suggest that healthcare providers may find it beneficial to provide structured, step-by-step information at diagnosis rather than one-time technical explanations. Our review detailed how braces/harnesses technicalize daily parenting tasks and reduce parent–child intimacy. These findings align with Lenders et al. (23), who found that brace treatment success is associated with family support systems. De Pellegrin et al. (5) also noted that non-surgical treatment requires high family cooperation. Our review further reveals that the emotional costs of this “cooperation”—persistent anxiety, loss of intimacy—may be underestimated by clinicians. We identified three specific patterns of support failure: mismatches in timing, content, and credibility. This aligns with Alnowaishiri et al. (24) and Almatari et al. (25), both finding that caregivers generally lack knowledge and credible information about DDH. Theunissen et al. (19) specifically studied information provision strategies, emphasizing the need for staged, personalized, multi-format information. Our review supports this conclusion and further highlights the importance of economic support, consistent with Sawamura et al. (26) and Lei et al. (27). This review found that caregivers gradually adapt through skill learning and meaning-making. However, confidence in this finding is low, based primarily on a single case study from 2003. This highlights a major gap in the current literature: our understanding of the long-term adaptation process of caregivers is very limited. Future longitudinal, in-depth qualitative studies are needed to explore this process. Based on the findings, the following practice implications may be considered: (1) providing step-by-step, understandable information at diagnosis to avoid information overload; (2) considering whether specialist nurses, or equivalent members of the multidisciplinary team, could serve as a bridge between families and physicians, offering practical guidance for daily care; (3) signposting families to vetted online support resources and, where appropriate, peer support networks; (4) it may be helpful to inform families of potential economic burdens before treatment begins, including equipment, clothing, and indirect costs; (5) considering regular assessment of caregiver emotional status and adaptation progress, with offers of psychological support when appropriate.

## 5 Limitations

Several limitations should be acknowledged. First, although a systematic search was conducted, only English-language publications were included, and the geographical scope was limited to Canada, Australia, the Netherlands, and China, which may restrict the transferability of findings to other cultural and healthcare contexts. Second, the included studies exhibited considerable heterogeneity in study design, ranging from in-depth qualitative interviews to large-scale surveys with open-ended questions. This methodological diversity, while enriching the breadth of perspectives, may have introduced variability in the depth and richness of the data available for synthesis. Third, despite the overall sample size being substantial, the number of studies employing in-depth qualitative interviews—which are better suited to capturing nuanced lived experiences—remains limited (only four). This may have constrained the depth of insight into certain aspects of caregivers’ experiences, particularly the nuanced mechanisms of adaptation. Fourth, the finding on adaptation (Theme 4) relies heavily on a single case study from 2003 (16), which severely limits the confidence and generalizability of this finding. Fifth, regarding the study selection process, although the inter-rater reliability reached a substantial level (Cohen’s *κ* = 0.714), no additional calibration exercise was conducted prior to screening. While this may introduce some minor variability, the two reviewers had thoroughly discussed and pilot-tested the inclusion criteria beforehand, and the achieved κ value suggests that any potential impact on the overall findings is likely minimal.

## 6 Conclusion

This review summarizes the qualitative evidence on caregiving experiences of primary caregivers of children with DDH. Four main descriptive themes were identified: a challenging and often delayed diagnostic process; treatment devices complicate daily parenting tasks; inadequate or ill-timed informational and practical support; and gradual adaptation and skill development over time. These findings indicate that caring for a child with DDH is not merely a medical journey but a profound family experience that reshapes parental identity, daily routines, and family dynamics. Importantly, the evidence base for the adaptation process is weak, relying primarily on a single case study from 2003. This highlights an urgent direction for future research: more longitudinal, in-depth qualitative studies are needed to fully understand how caregivers adapt over time. Healthcare providers would benefit from recognizing the multifaceted nature of caregiver burden and could consider offering timely, practical, and empathetic support that addresses informational needs, facilitates peer connections, and acknowledges the adaptive work families undertake. Future research and clinical practice may consider adopting family-centered evaluation frameworks that capture what truly matters to those who provide daily care, ensuring that interventions are judged not only by clinical efficacy but also by their impact on the entire family unit.

## Data Availability

The original contributions presented in the study are included in the article/Supplementary material, further inquiries can be directed to the corresponding author.

## References

[1] Shu B Zhang S Gao J Wang L Wang X. Deciphering the past status and future tendency: a comprehensive scientometric study on developmental dysplasia of the hip. J Orthop Surg Res. (2024) 19:853. doi: 10.1186/s13018-024-05358-8, 39702394 PMC11656727

[2] Chen SS Han XH Gu FW. Analysis of ultrasound screening for developmental dysplasia of the hip in 6189 infants aged 0–6 months. J Clin Ultrasound. (2025) 54:854–63. doi: 10.1002/jcu.70140 41246944

[3] Alrashdi N Alotaibi M Alharthi M Kashoo F Alanazi S Alanazi A . Incidence, prevalence, risk factors, and clinical treatment for children with developmental dysplasia of the hip in Saudi Arabia. A systematic review. J Epidemiol Glob Health. (2024) 14:549–60. doi: 10.1007/s44197-024-00217-5, 38483754 PMC11444034

[4] Kim AM Kim KD Covey CM. Developmental dysplasia of the hip. Am Fam Physician. (2025) 112:546–52. 41252837

[5] De Pellegrin M Sarzana M Emedoli D Romeni S Marcucci L Guindani N. The influence of non-surgical treatment on walking age in children with severe developmental dysplasia of the hip. J Child Orthop. (2025) 19:446–54. doi: 10.1177/18632521251390250, 41262549 PMC12623227

[6] Gibbard M Zivkovic I Jivraj B Schaeffer E Robillard JM Mulpuri K . A global survey of patient and caregiver experiences throughout Care for Developmental Dysplasia of the hip. J Pediatr Orthop. (2021) 41:e392–7. doi: 10.1097/BPO.0000000000001813, 34096547 PMC8183474

[7] Harry A Johnston C Twomey S Wakely L. A survey of parents' and Carers' perceptions of parenting a child with developmental dysplasia of the hip. Pediatr Phys Ther. (2022) 34:328–33. doi: 10.1097/PEP.0000000000000917, 35639555

[8] Craven J Wise H Perry DC Plumpton C. Parental preferences for brace weaning in developmental dysplasia of the hip: a discrete choice experiment. Bone Jt Open. (2025) 6:1581–7. doi: 10.1302/2633-1462.612.BJO-2025-0273.R1, 41371274 PMC12695216

[9] Craven J O'Malley O Perry DC. Development of a family-centred core outcome set for infants with developmental dysplasia of the hip treated with a brace. Bone Jt Open. (2025) 6:21–5. doi: 10.1302/2633-1462.61.BJO-2024-0186, 39753147 PMC11698604

[10] Wakely L Easey P Leys J Johnston C. Exploring the lived experience of parenting a child with developmental dysplasia of the hip. Phys Occup Ther Pediatr. (2021) 41:503–14. doi: 10.1080/01942638.2020.1867694, 33557686

[11] Theunissen W van der Steen MC van Veen MR . Parental experiences of children with developmental dysplasia of the hip: a qualitative study. BMJ Open. (2022) 12:e62585. doi: 10.1136/bmjopen-2022-062585 PMC9511546 36153020

[12] McHugh ML. Interrater reliability: the kappa statistic. Biochem Med (Zagreb). (2012) 22:276–82. doi: 10.11613/BM.2012.031, 23092060 PMC3900052

[13] Aromataris E Lockwood C Porritt K Pilla B Jordan Z, editors. JBI Manual for Evidence Synthesis. JBI; (2024). Available online at: https://synthesismanual.jbi.global (Accessed February 12, 2025).

[14] Lockwood C Porritt K Munn Z. . Systematic Reviews of Qualitative Evidence[EB/OL]. Available online at: https://jbi.global/ (Accessed February 12, 2025).

[15] Lewin S Booth A Glenton C Munthe-Kaas H Rashidian A Wainwright M . Applying GRADE-CERQual to qualitative evidence synthesis findings: introduction to the series. Implement Sci. (2018) 13:2. doi: 10.1186/s13012-017-0688-3, 29384079 PMC5791040

[16] Chao M Chiang VC. Impact on and coping behaviours of a Chinese mother with a child suffering from developmental dysplasia of the hip. J Orthop Nurs. (2003) 7:176–83. doi: 10.1016/j.joon.2003.08.001

[17] Poole C. Exploring the Experiences of Parents Caring for an Infant with Developmental Dysplasia of the Hip (DDH): Interpretative Phenomenological Analysis (2019).

[18] Grzybowski G Bliven E Wu L Schaeffer EK Gibbard M Zomar BO . Caregiver experiences using orthotic treatment options for developmental dysplasia of the hip in children. J Pediatr Orthop. (2023) 43:105–10. doi: 10.1097/BPO.0000000000002312, 36607922 PMC9812410

[19] Theunissen W Van der Steen MC Van Veen MR Van Douveren FQMP Witlox MA Tolk JJ. Strategies to optimize the information provision for parents of children with developmental dysplasia of the hip. Bone Jt Open. (2023) 4:496–506. doi: 10.1302/2633-1462.47.BJO-2023-0072.R1 37402475 PMC10319458

[20] Craven J Theunissen W O'Malley O Winson DMG Morley E Asher M . A family-centred core outcome set for infants with developmental dysplasia of the hip undergoing brace treatment. Bone Joint J. (2025) 107-B:973–8. doi: 10.1302/0301-620X.107B9.BJJ-2025-0059.R1 40887055

[21] Gönen E Egici MT Gönen E. Knowledge and awareness of family physicians as key stakeholders regarding developmental dysplasia of the hip and the nationwide screening program. Acta Orthop Traumatol Turc. (2025) 59:49–57. doi: 10.5152/j.aott.2025.24034, 40338009 PMC11992945

[22] Zhang Y Fu C Junxia W Yang L Qin Z. Efficacy and safety of various regional nerve blocks for postoperative analgesia in paediatric patients undergoing developmental dysplasia of the hip surgery: a protocol for systematic review and network meta-analysis. BMJ Open. (2024) 14:e89194. doi: 10.1136/bmjopen-2024-089194 PMC11667397 39806655

[23] Lenders J Rajaram P Henry BW Gornitzky AL Li Y. Public insurance status is associated with lower bracing treatment success in infants with developmental dysplasia of the hip. J Pediatr Orthop. (2025) 45:e599–605. doi: 10.1097/BPO.0000000000002925, 40353607

[24] Alnowaishiri KA Khader JE Alkanderi WK Alshiyab SM Jaber KA Samarah OQ. Knowledge, attitudes, and perceptions of developmental dysplasia of the hip among pregnant women in Jordan. Saudi Med J. (2025) 46:831–5. doi: 10.15537/smj.2025.46.7.20241131, 40628442 PMC12251593

[25] Almatari AH Alhazmi NF Jafar HM Althagafi AA Qasim OM Alghamdi FA . Maternal perceptions and awareness regarding developmental dysplasia of the hip in children among mothers and pregnant women in Makkah City, Saudi Arabia. J Family Med Prim Care. (2024) 13:4041–6. doi: 10.4103/jfmpc.jfmpc_72_24, 39464946 PMC11504760

[26] Sawamura K Kitoh H Matsushita M Mishima K Kamiya Y Imagama S. Quality of life in adult patients with developmental dysplasia of the hip who were treated for hip dislocation during childhood. J Pediatr Orthop B. (2025) 34:38–43. doi: 10.1097/BPB.0000000000001173, 38451811

[27] Lei Y Yan J Wenjuan S Weili S Qianyi X Lingyan F. Caregivers' care experiences of children with developmental dislocation of the hip in Tibet, China: a convergent mixed-methods study. Front Pediatr. (2025) 13:1561246. doi: 10.3389/fped.2025.1561246, 40416440 PMC12098034

