# Supplementary material for: Caregivers’ experiences of caring for children with developmental dysplasia of the hip: a qualitative descriptive review
Source: Front Med (Lausanne). 2026 May 14;13:1772584. doi: 10.3389/fmed.2026.1772584 (PMC13215836; doi:10.3389/fmed.2026.1772584)
Supplement: Supplementary file 1 [file Table_1.DOCX]

Appendix I: Search strategy for PubMed (MEDLINE)

Database: PubMed (MEDLINE)

| Set # |  | Results |
| --- | --- | --- |
| 1 | “Developmental Dysplasia of the Hip”[Mesh] OR “Hip Dislocation, Congenital”[Mesh] OR “Hip Dislocation, Development*”[tiab] OR “Development* Hip Dislocation” [tiab] OR “Dislocation, Development* Hip”[tiab] OR “Development* Hip Dysplasia”[tiab] OR “Dysplasia, Development* Hip”[tiab] OR “Hip Dysplasia, Development*” OR “Congenital Hip Dislocation” OR “Dislocation, Congenital Hip” OR “Hip Dislocation, Congenital” OR “Congenital Hip Displacement” OR “Displacement, Congenital Hip” OR “Hip Displacement, Congenital” OR “Congenital Hip Dysplasia” OR “Dysplasia, Congenital Hip” OR “Hip Dysplasia, Congenital” OR “Hip, Dislocation Of, Congenital” OR “Congenital Dysplasia Of The Hip” OR “Dislocation Of Hip, Congenital” OR “Hip Dysplasia, Congenital, Nonsyndromic” OR DDH | 12519 |
| 2 | father OR men OR dad* OR paternal OR male OR mother* OR maternal OR parent* OR caregiver* OR (carer)) OR (family members)) OR (relatives)) OR (informal carers)) OR (family)) OR (home) | 12，549，312 |
| 3 | (((((((((((experiences) OR (perceptions)) OR (attitudes)) OR (views)) OR (feelings)) OR (qualitative)) OR (perspective)) OR (caring)) OR (nursing)) OR (care)) OR (parenting)) OR (living) | 6，852，270 |
| 4 | #1 AND #2 AND #3 | 1126 |
